# Supplementary material for: Developmental and molecular contributions to contextual fear memory emergence in mice
Source: Neuropsychopharmacology. 2024 Mar 5;49(9):1392–401. doi: 10.1038/s41386-024-01835-0 (PMC11251045; doi:10.1038/s41386-024-01835-0)
Supplement: Supplementary file 1 — Supplemental Material [file 41386_2024_1835_MOESM1_ESM.pdf]

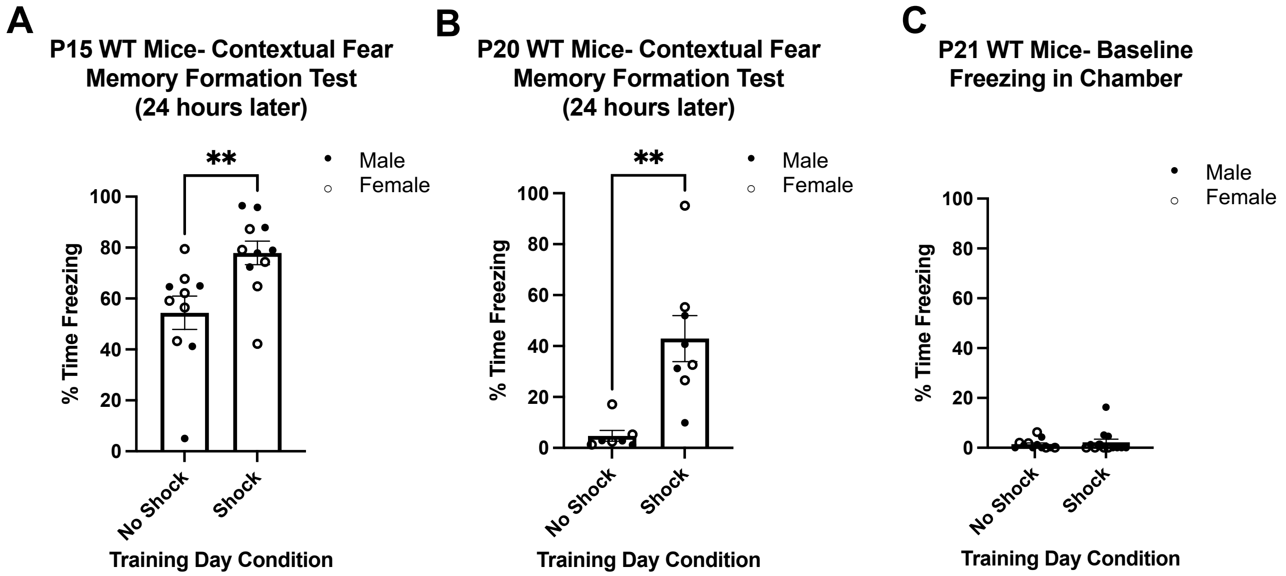

Fig. S1: P15 and P20 WT mice exhibit contextual fear memory formation capabilities, and P21 mice exhibit no baseline freezing differences.

A Quantification of the percentage time freezing on testing day of No Shock (n = 10) and Shock (n = 11) mice trained on P15 and tested 1 d later. The Shock group exhibited significantly more freezing than the No Shock group (**p < .01). **B** Quantification of the percentage time freezing on testing day of No Shock (n = 7) and Shock (n = 8) mice trained on P20 and tested 1 d later. The Shock group exhibited significantly more freezing than No Shock (**p < .01). **C** Quantification of the percentage time freezing during the initial 2-min exposure to the chamber on training day and prior to any shocks administered (baseline freezing) on P21 (No Shock group: n = 12; Shock group: n = 13). There were no significant differences found in freezing responses between the two groups (p = .3571).

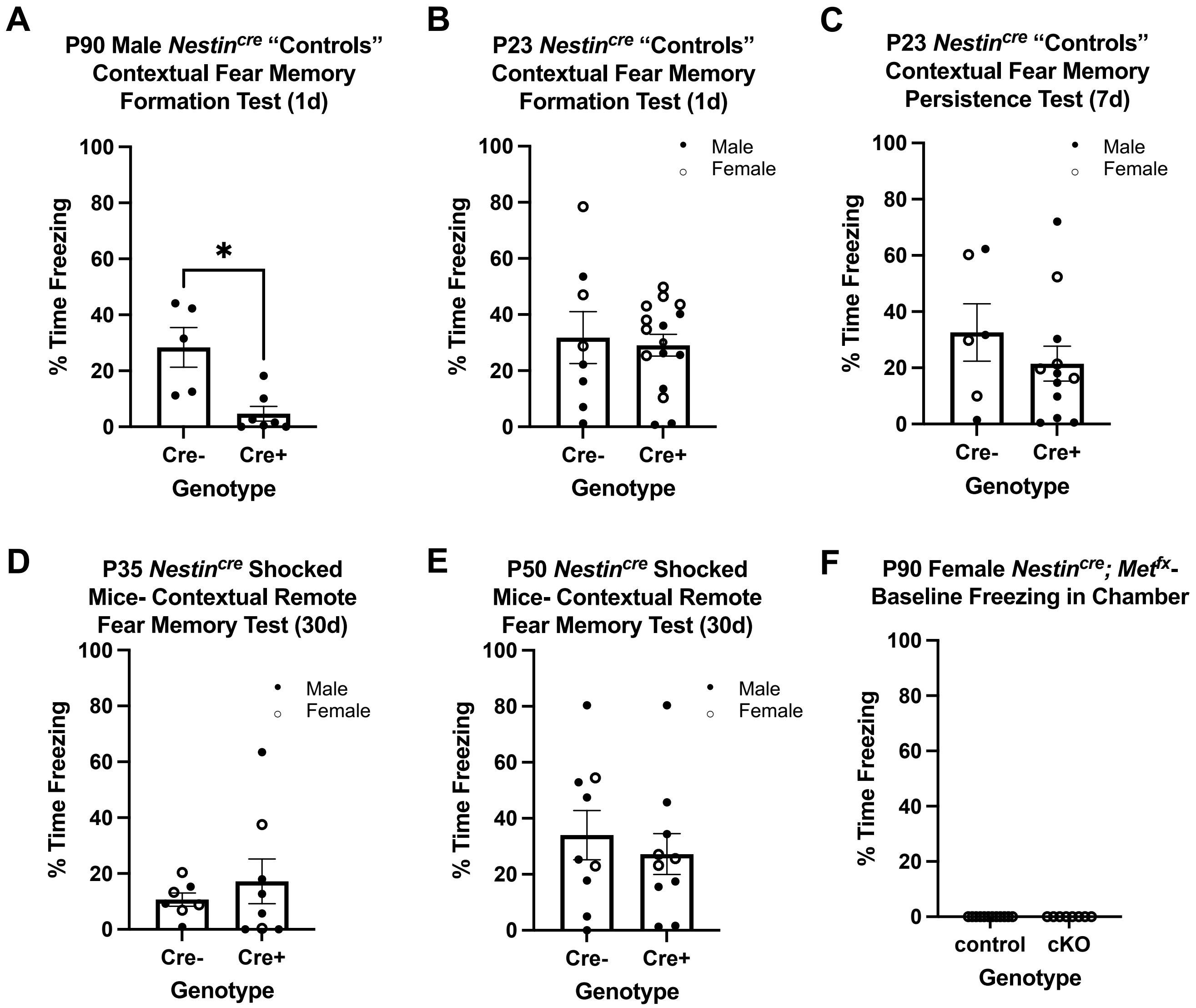

Fig. S2: There are no genotype differences in control *Nestin*^{Cre} mice during development, unlike in males in adulthood. There is no baseline freezing in P90 females. Quantification of the percentage time freezing on testing day between Cre- and Cre+, control mice. **A** Cre+ control male mice (n = 7) trained on P90 and tested 1 d later exhibit significantly less freezing (p = .0152) than Cre- control male mice (n = 5). No significant differences were determined during development: **B** Trained on P23 and tested 1 d later (p = .7501; Cre-: n = 8; Cre+: n = 16) **C** Trained on P23 and tested 7 d later (p = .4680; Cre-: n = 6; Cre+: n = 12) **D** Trained on P35 and tested 30 d later (p = .5077; Cre-: n = 7; Cre+: n = 8) or **E** Trained on P50 and tested 30 d later (p = .6277; Cre-: n = 9; Cre+: n = 10). **F** There is no baseline freezing on conditioning day in P90 female control (n = 12) or cKO (n = 8) mice.
